# MicroRNA-93 Promotes Epithelial–Mesenchymal Transition of Endometrial Carcinoma Cells

**DOI:** 10.1371/journal.pone.0165776

**Published:** 2016-11-09

**Authors:** Shuo Chen, Xi Chen, Kai-Xuan Sun, Yin-Ling Xiu, Bo-Liang Liu, Miao-Xiao Feng, Xiu-Bo Sang, Yang Zhao

**Affiliations:** Department of Gynecology, The First Affiliated Hospital of China Medical University, Shenyang, Liaoning, China; Beijing Cancer Hospital, CHINA

## Abstract

MicroRNA-93, derived from a paralog (miR-106b-25) of the miR-17-92 cluster, is involved in the tumorigenesis and progression of many cancers such as breast, colorectal, hepatocellular, lung, ovarian, and pancreatic cancer. However, the role of miR-93 in endometrial carcinoma and the potential molecular mechanisms involved remain unknown. Our results showed that miR-93 was overexpressed in endometrial carcinoma tissues than normal endometrial tissues. The endometrial carcinoma cell lines HEC-1B and Ishikawa were transfected with miR-93-5P, after which cell migration and invasion ability and the expression of relevant molecules were detected. MiR-93 overexpression promoted cell migration and invasion, and downregulated E-cadherin expression while increasing N-cadherin expression. Dual-luciferase reporter assay showed that miR-93 may directly bind to the 3′ untranslated region of forkhead box A1 (*FOXA1*); furthermore, miR-93 overexpression downregulated FOXA1 expression while miR-93 inhibitor transfection upregulated FOXA1 expression at both mRNA and protein level. In addition, transfection with the most effective FOXA1 small interfering RNA promoted both endometrial cancer cell migration and invasion, and downregulated E-cadherin expression while upregulating N-cadherin expression. Therefore, we suggest that miR-93 may promote the process of epithelial–mesenchymal transition in endometrial carcinoma cells by targeting *FOXA1*.

## Introduction

Endometrial carcinoma is one of the most common tumors of the female reproductive system [1]. The incidence of endometrial carcinoma has dramatically increased over the past decades. Recurrence and metastasis are important causes of death in patients with endometrial cancer, and some studies have found that tumor invasion and metastasis are closely related to epithelial–mesenchymal transition (EMT) [2–6]. EMT is an intricate process where cancer cells demonstrate the loss of polarity and cell phenotype alteration from epithelial to mesenchymal morphology and it is considered a prerequisite for the typical tumor phenotypes of upregulated angiogenesis, enhanced cell motility, and extracellular matrix invasion [7–8]. In addition, it has been indicated that, in endometrial carcinoma, metastasis and recurrence are closely related with EMT [9–10].

MicroRNA (miRNA), a class of naturally occurring, small noncoding RNA, bind to the 3′ untranslated region (3′UTR) of target genes to downregulate their expression at post-transcription level by inducing the degradation of the targeted mRNA or inhibiting translation of the targeted mRNA and subsequently regulating diverse basic cellular functions such as proliferation, migration, invasion, and the EMT process [11–15]. In ovarian cancer, Ras homolog family member C (RhoC) overexpression promotes EMT and increased migration and invasion ability [16]. In our previous study, we found that miR-93 downregulated RhoC to inhibit cell migration and invasion in ovarian cancer [17]. However, the role of miR-93 in endometrial carcinoma and the potential molecular mechanisms involved remain unknown. This is the first study to investigate role of miR-93 in influencing the migration and invasion ability of endometrial carcinoma cells via EMT. We found that miR-93 promoted endometrial carcinoma cell EMT, migration, and invasion.

## Materials and Methods

### Cell culture and transfection

The endometrial carcinoma cell lines Ishikawa was purchased from Nanjing Keygen Biotech (Nanjing, China) and HEC-1B was purchased from the China Center for Type Culture Collection (CCTCC, Wuhan, China). The Ishikawa cells were cultured in RPMI 1640 medium; the HEC-1B cells were maintained in Dulbecco’s modified Eagle’s medium supplemented with 10% fetal bovine serum (FBS) and 100 U/mL penicillin / streptomycin. The two cell lines were propagated at 37°C in a humidified atmosphere of 5% CO2 in air. MiR-93 mimics, non-targeting control mimic, anti-miR-93 mimics, anti-miR-Control, three forkhead box A1 (FOXA1) small interfering RNA (siRNA) (siFOXA1, siFOXA1-1, siFOXA1-2), or scramble siRNA (Mock) (Sigma, Shanghai, China) were transiently transfected into the cell lines using Lipofectamine 2000 (Invitrogen, Carlsbad, USA) according to the manufacturer’s protocol. For would healing and Transwell assay, the checkpoints was 24h after transfection, while 48h for western blot, RT-PCR and Immunofluorescence assays.

### Wound healing assay

Cells were seeded at 1.0 × 10^6^ cells/well in 6-well culture plates. After they had grown to confluence, the cell monolayer was scraped with a pipette tip (200 μL) to create a scratch, washed thrice with phosphate-buffered saline (PBS), and cultured in FBS-free medium. The cells were photographed at 0 and 24h, and the scratch area was measured using Image J software (National Institutes of Health, Bethesda, MD, USA). The migration rate = (area of original wound − area of wound at different times)/area of original wound × 100%.

### Cell invasion assay

For the invasion assay, 5 × 10^4^ cells were resuspended in serum-free medium and seeded into the top chambers of Matrigel-coated Transwell inserts (BD Bioscience, San Jose, CA, USA). The bottom compartment of the chamber contained complete medium as a chemoattractant. After 24-h incubation, cells on the top surface of the membrane were removed slightly by the dry cotton swabs, and cells on the bottom surface of the membrane were washed with PBS, fixed in formaldehyde, and stained with crystal violet and counted in five representative microscopic fields under Olympus fluorescence microscope (Tokyo, Japan) to quantify the extent of invasion, and three independent experiments were performed.

### Immunofluorescence

Cells were cultured on glass coverslips, fixed with 4% formaldehyde for 10 min, and permeabilized with 0.2% Triton X-100 in PBS for 30 min at room temperature. After washing three times, 1% bovine serum albumin (BSA) was used for blocking. After 1 h, the cells were incubated with rabbit anti-human primary antibodies to E-cadherin and N-cadherin (Santa Cruz Biotechnology, Santa Cruz, CA, USA) at 1:50 dilution at 4°C overnight. Thereafter, the cells were washed thrice with Tris-buffered saline with Tween 20 (TBST) and further incubated with fluorescein isothiocyanate (FITC)-labeled secondary antibody (anti-rabbit, 1:100, Santa Cruz Biotechnology, Santa Cruz, CA, USA) at room temperature for 2 h. The nuclei were stained with 1 μg/mL diaminophenylindole (DAPI, 1:100; Sigma-Aldrich, St. Louis, MO, USA) for 5 min at 37°C. The coverslips were then mounted with SlowFade Gold Antifade Reagent (Invitrogen) and observed under a confocal laser microscope (Olympus, Tokyo, Japan).

### Western blot

Western blotting was performed using a Bio-Rad protein assay kit (Hercules, CA, USA). Denatured proteins were separated by sodium dodecyl sulfate–polyacrylamide gel electrophoresis (SDS-PAGE) on 10% acrylamide gels, and then transferred to Hybond membranes (Amersham, Freiburg, Germany). The membranes were blocked overnight in 5% fat-free milk in TBST. The membranes were incubated with primary antibodies against E-cadherin, N-cadherin, FOXA1, and glyceraldehyde-3-phosphate dehydrogenase (GAPDH) (Santa Cruz Biotechnology, Santa Cruz, CA, USA) overnight at 4°C at 1:500 or 1:2000 (GAPDH) dilutions. Thereafter, the membranes were washed thrice in TBST, and further incubated with secondary antibody (1:5000) at room temperature for 2 h. After applying electrochemiluminescence (ECL) Plus detection reagents (Santa Cruz Biotechnology, Santa Cruz, CA, USA), the protein bands were visualized using X-ray film (Fujifilm, Tokyo, Japan).

### Real-time reverse transcription–PCR (RT-PCR)

TRIzol reagent (Takara, Shiga, Japan) was used to isolate RNA from the tissues or cells. The RNA was reverse-transcribed to complementary DNA (cDNA) using an avian myeloblastosis virus transcriptase and random primers (Takara) according to the manufacturer’s protocol. The cDNA was amplified by RT-PCR with a SYBR Premix Ex Taq II kit (Takara). Denaturation was at 94°C for 30 sec, annealing was at 51°C for 60 sec, and extension was at 72°C for 60 sec. The data analysis was performed according to the sample threshold cycle (Ct) value from three independent experiments. 18sRNA was set as the inner control.

### Dual-luciferase reporter assay

HEK293T cells were used to perform the dual-luciferase reporter assay. *FOXA1* 3′UTR clones or mutant clones were cotransfected with miR-93 using Lipofectamine 2000 24 h after plating. PGL-TK vector expressing *Renilla* luciferase was used as the transfection control. Luciferase activity was measured using a Dual-Luciferase Reporter Assay System (Promega, Madison, WI, USA) according to the manufacturer’s protocol. For each transfection, the luciferase activity was averaged from three replicates.

### Endometrial carcinoma specimens

57 endometrial carcinomas (ECs) and 12 normal endometrial specimens were collected from patients who had undergone surgical resection at the Department of Gynecology of the First Affiliated Hospital of China Medical University (Shenyang, Liaoning, China). None of the patients had preoperative chemotherapy or radiotherapy. Informed written consent was obtained from all participants and the research protocol was approved by the China Medical University Ethics Committee (No: 2016-32-2).

### Statistical analysis

The results were analyzed using a double-sided Student’s *t*-test to compare the two independent groups and ANOVA (Analysis of Variance) with post-test to compare the three independent groups. Statistical evaluation was performed using SPSS v. 17.0 software (Chicago, IL, USA). A P-value < 0.05 was considered statistically significant.

## Results

### MiR-93 expression in endometrial carcinoma tissues and normal endometrial specimens

**Fig 1:**
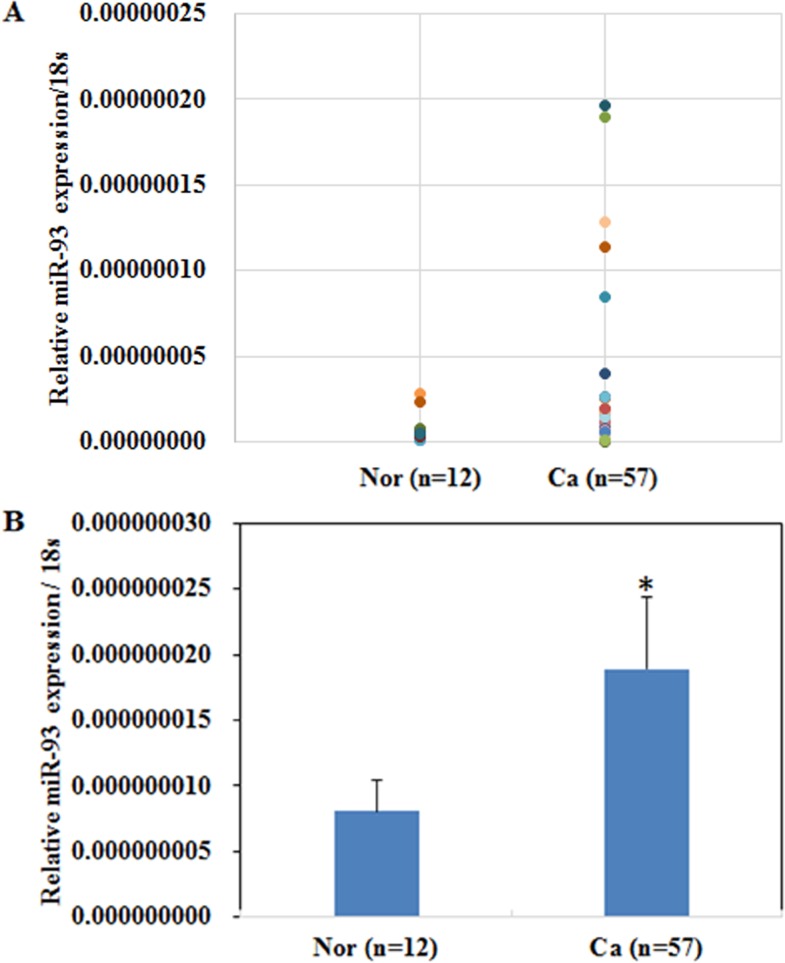
MiR-93 expression in endometrial carcinoma tissues and normal endometrial specimens. Expression levels of miR-93 in both endometrial carcinoma samples and normal samples were analyzed by RT-PCR **(A)**. MiR-93 was highly expressed in EC tissue than in normal samples **(B)**. *P < 0.05.

Expression levels of miR-93 in both endometrial carcinoma samples and normal samples were analyzed by RT-PCR (Fig 1A). As shown in Fig 1, miR-93 was highly expressed in EC tissue than in normal samples (P < 0.05; Fig 1B).

### MiR-93 transfection promoted endometrial carcinoma cell migration and invasion

**Fig 2:**
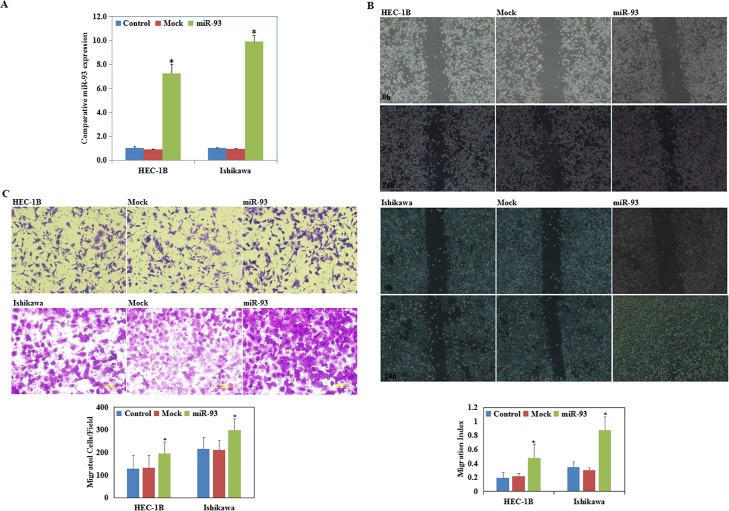
MiR-93 transfection promoted endometrial carcinoma cell migration and invasion. Following miR-93 transfection, miR-93 levels were significantly increased **(A)**, endometrial carcinoma cell lines exhibited faster migration **(B)**, and stronger invasion **(C)** compared with the control and mock cells 24 hours after transfection. Three representative microscopic fields were photographed to quantify the extent of invasion and migration, and three independent experiments were performed. Results are representative of three separate experiments; data are expressed as the mean ± standard deviation, *P < 0.05.

Endometrial carcinoma cells were transfected with miR-93 mimics to upregulate miR-93 expression. Quantitative RT-PCR (qRT-PCR) revealed significantly increased miR-93 levels (P < 0.05; Fig 2A).

The wound healing assay showed that cells transfected with miR-93 presented faster closing of the scratch wound as compared with the control cells (P < 0.05; Fig 2B). The Transwell assays showed that cells transfected with miR-93 had significantly increased invasive ability as compared with the control cells (P < 0.05; Fig 2C).

### MiR-93 transfection downregulated E-cadherin expression and upregulated N-cadherin expression

**Fig 3:**
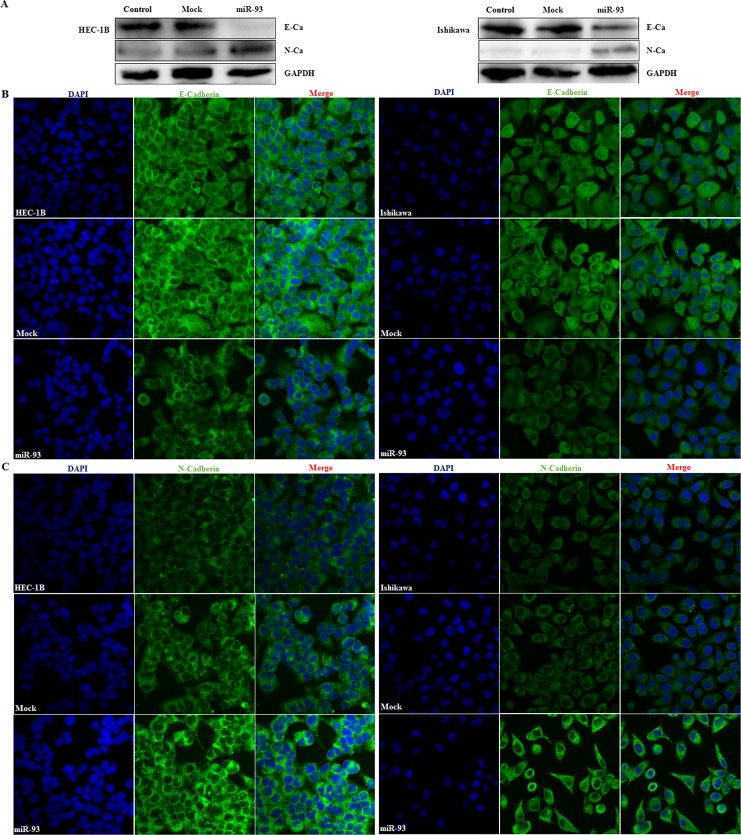
MiR-93 transfection downregulated expression of E-cadherin and upregulated expression of the N-cadherin. After miR-93 transfection, E-cadherin was downregulated and N-cadherin was upregulated in Western blot assay (A). Besides, Immunofluorescence assay was used to determine subcellular localization of E-cadherin and N- cadherin (B & C).

Following miR-93 transfection, western blotting revealed significantly decreased E-cadherin expression and significantly increased N-cadherin expression (Fig 3A). We further investigated the distribution of E-cadherin and N-cadherin using cellular immunofluorescence (IF). Cellular IF demonstrated that E-cadherin was predominantly located at the membrane of normal and mock transfected endometrial cancer cells, while lack of periphery distribution was observed in miR-93 transfection cells (Fig 3B), while miR-93 transfection induced N-cadherin distribution at the membrane and cytoplasm (Fig 3C).

### MiR-93 transfection downregulated FOXA1 expression

**Fig 4:**
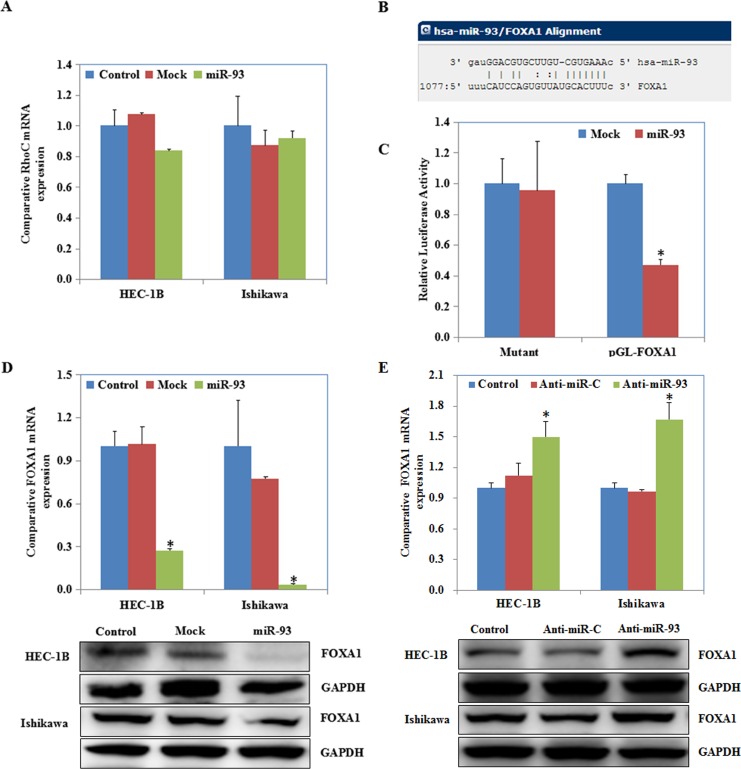
MiR-93 transfection downregulated expression of FOXA1. After miR-93 transfection the expression of RhoC had no significant differences (A) but FOXA1 was downregulated (D). According to a prediction website and Luciferase reporter assays demonstrated miR-93 could targets the 3’UTR of FOXA1 directly (B & C). MiR-93 inhibitor transfection induced FOXA1 expression (E).

RhoC expression was not significantly different after the endometrial carcinoma cells had been transfected with miR-93 (Fig 4A). According to the microRNA.org prediction website, the *FOXA1* 3′UTR contains a complementary sequence of miR-93 (Fig 4B). The luciferase reporter assays demonstrated that miR-93 significantly decreased the relative luciferase activity of the wild-type *FOXA1* 3′UTR as compared with the mutant *FOXA1* 3′UTR (P < 0.05; Fig 4C). RT-PCR and western blotting showed that miR-93 transfection reduced FOXA1 expression (Fig 4D), while miR-93 inhibitor transfection induced FOXA1 expression (Fig 4E).

### SiFOXA1 transfection promoted endometrial carcinoma cell migration and invasion

**Fig 5:**
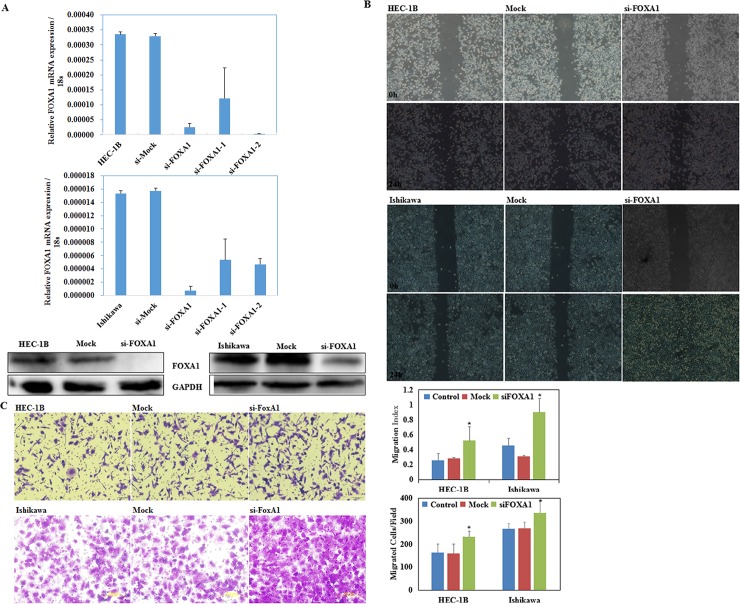
siFOXA1 transfection promoted endometrial carcinoma cell migration and invasion. After siFOXA1 transfection, the expression levels of FOXA1 decreased significantly (A). Endometrial carcinoma cell lines exhibited faster migration **(B)**, and stronger invasion **(C)** compared with the control and mock cells 24 hours after transfection. Three representative microscopic fields of each well (n = 3) were documented to quantify the extent of invasion and migration, and three independent experiments were performed. Data are expressed as the mean ± standard deviation, *P < 0.05.

Endometrial carcinoma cells were transfected with FOXA1 siRNAs (siFOXA1, siFOAX1-1, and siFOXA1-2) to downregulate FOXA1 expression. After transfection, we analyzed FOXA1 expression using RT-PCR and western blotting, and found significantly decreased FOXA1 expression levels (P < 0.05; Fig 5A), and siFOXA1 was the most effective siRNA in both two cell lines, thus it was used for the following experiments.

The wound healing assay showed that cells transfected with siFOXA1 presented faster closing of the scratch wound as compared with the control (P < 0.05; Fig 5B). The Transwell assay showed that cells transfected with siFOXA1 demonstrated stronger invasive ability as compared with the control (P < 0.05; Fig 5C).

### SiFOXA1 transfection downregulated E-cadherin expression and increased N-cadherin expression

**Fig 6:**
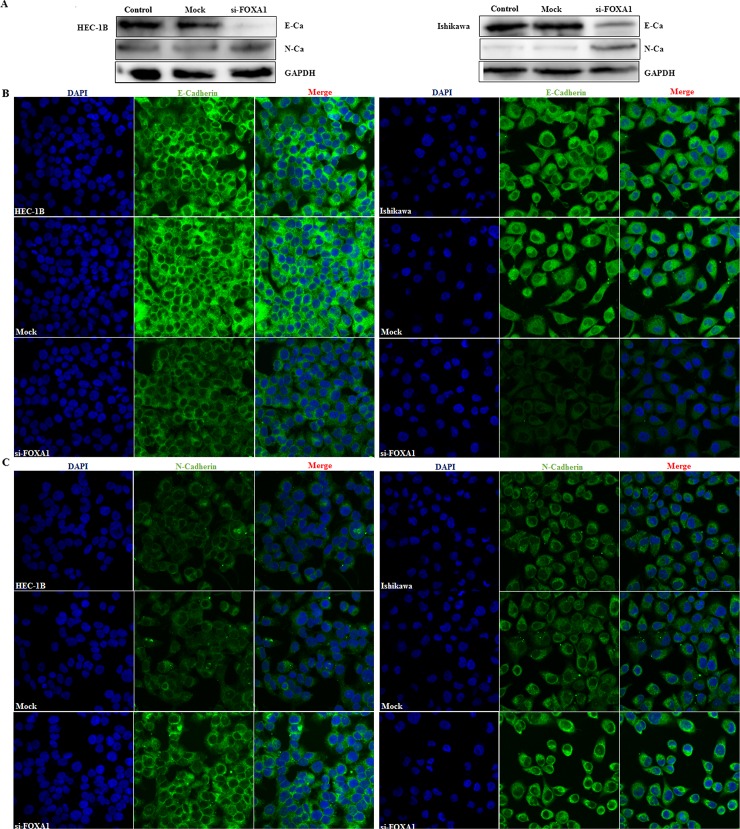
siFOXA1 transfection downregulated expression of E-cadherin and increased expression of the N-cadherin. After siFOXA1 transfection, E-cadherin was downregulated and N-cadherin was upregulated in Western blot assay (A). Besides, Immunofluorescence assay was used to determine subcellular localization of E-cadherin and N- cadherin (B & C).

Following siFOXA1 transfection, western blotting revealed decreased E-cadherin expression and increased N-cadherin expression (Fig 6A). We further investigated the distribution of E-cadherin and N-cadherin using cellular immunofluorescence (IF). Cellular IF demonstrated that E-cadherin was predominantly located at the membrane of normal and mock transfected endometrial cancer cells, while lack of periphery distribution was observed in siFOXA1 transfection cells (Fig 6B), while siFOXA1 transfection induced N-cadherin distribution at the membrane and cytoplasm (Fig 6C).

## Discussion

It is well known that invasion and metastasis are important poor prognostic factors in malignancy. EMT is considered an important step in tumor invasion and metastasis, involving the transformation of epithelial cells to mesenchymal cells. Through EMT, the transformed epithelial cells gain mesenchymal traits that appear to contribute to metastasis. Individual cancer cells with a mesenchymal phenotype can cross the endothelial barriers and enter the blood and lymphatic circulation [18]. A critical molecular feature of this process is the downregulation of E-cadherin, an epithelial cell marker, and the upregulation of N-cadherin, an interstitial cell marker [19–21].

In other malignant tumors, many reports state that miRNAs can influence tumor invasion and metastasis by participating in the EMT process [22–26]. In ovarian cancer, RhoC overexpression promotes EMT, invasion, and metastasis, and RhoC knockout reverses the promoter role of vascular endothelial growth factor (VEGF) and transforming growth factor (TGF) in EMT [16]. In a previous study, we found that miR-93 downregulates RhoC to inhibit migration and invasion in ovarian cancer [17]. However, the present study derived opposite results in that miR-93 was highly expressed in endometrial carcinoma tissues and, what’s more, miR-93 overexpression promoted endometrial carcinoma cell migration and invasion, and downregulated E-cadherin expression and increased N-cadherin expression, while RhoC expression in endometrial carcinoma cells was unchanged after miR-93 transfection. MiRNAs and their specific targets are dependent on specific cellular environments [27]. Therefore, we guess that the role of miR-93 in endometrial cancer may differ from its role in ovarian cancer and that it may plays a promoter role in EMT, migration, and invasion in endometrial carcinoma.

Next, we analyzed the potential molecular mechanism involved. According to the TargetScan and miRanda prediction websites, the 3′UTR of *FOXA1* mRNA contains the complementary sequence of miR-93, which was confirmed by the luciferase reporter assays. MiR-93 transfection in the endometrial cancer cells downregulated FOXA1 expression while miR-93 inhibitor transfection upregulated FOXA1 expression significantly. FOXA1, which belongs to the FOX family of transcription factors, is a negative regulator of epithelial-mesenchymal transition [28–29]. FOXA1 suppresses EMT in pancreatic cancer [30], prostate cancer [31–32] and breast cancer [33–35]. Wang et al. reported that FOXA1 expression was lower in endometrial carcinoma tissue than in normal endometrial tissues and was positively related to the degree of differentiation of endometrial carcinoma, and FOXA1 overexpression inhibited endometrial cancer cell proliferation [36]. Immunohistochemistry has shown that FOXA1 expression is negatively related to lymph node metastasis, and downregulating FOXA1 promotes endometrial cancer cell proliferation and migration [37]. We transfected endometrial carcinoma cells with siFOXA1 to downregulate FOXA1 expression, and found upregulated N-cadherin expression, downregulated E-cadherin expression, and promoted migration and invasion ability, which demonstrates that FOXA1 acts as a suppressor in EMT. Above all, we suggest that miR-93 promote EMT, migration, and invasion in endometrial carcinoma cells through downregulating FOXA1.

Our findings report for the first time that miR-93 promotes endometrial carcinoma cell EMT, migration, and invasion via targeting downregulation of FOXA1, which differs from its anti-oncogene role in ovarian cancer. Targeted therapy with miRNA in malignant tumors is tissue-specific; therefore miRNA selection should be made with care. Further preclinical studies are necessary to determine the role of the same miRNA in different malignant tumors.
